# *In Silico* Clinical Trial for Osteoporosis Treatments to Prevent Hip Fractures: Simulation of the Placebo Arm

**DOI:** 10.1007/s10439-024-03636-4

**Published:** 2024-11-22

**Authors:** Giacomo Savelli, Sara Oliviero, Antonino A. La Mattina, Marco Viceconti

**Affiliations:** 1 Department of Industrial Engineering, Alma Mater Studiorum - University of Bologna, Bologna, Italy; 2 Medical Technology Lab, IRCSS - Istituto Ortopedico Rizzoli, Via di Barbiano 1/10, 40136 Bologna, Italy

**Keywords:** Osteoporosis, Hip fracture, *In silico* clinical trial, Computational biomechanics, Retrospective validation, Multiscale model

## Abstract

Osteoporosis represents a major healthcare concern. The development of novel treatments presents challenges due to the limited cost-effectiveness of clinical trials and ethical concerns associated with placebo-controlled trials. Computational models for the design and assessment of biomedical products (*In Silico* Trials) are emerging as a promising alternative. In this study, a novel *In Silico* Trial technology (*BoneStrength*) was applied to replicate the placebo arms of two concluded clinical trials and its accuracy in predicting hip fracture incidence was evaluated. Two virtual cohorts (N = 1238 and 1226, respectively) were generated by sampling a statistical anatomy atlas based on CT scans of proximal femurs. Baseline characteristics were equivalent to those reported for the clinical cohorts. Fall events were sampled from a Poisson distribution. A multiscale stochastic model was implemented to estimate the impact force associated to each fall. Finite Element models were used to predict femur strength. Fracture incidence in 3 years follow-up was computed with a Markov chain approach; a patient was considered fractured if the impact force associated with a fall exceeded femur strength. Ten realizations of the stochastic process were run to reach convergence. Each realization required approximately 2500 FE simulations, solved using High-Performance Computing infrastructures. Predicted number of fractures was 12 ± 2 and 18 ± 4 for the two cohorts, respectively. The predicted incidence range consistently included the reported clinical data, although on average fracture incidence was overestimated. These findings highlight the potential of *BoneStrength* for future applications in drug development and assessment.

## Introduction

Osteoporosis (OP) represents a significant health concern worldwide, particularly among the ageing population. As life expectancy increases, especially in developed countries, the social and economic burden of osteoporosis is becoming more pronounced [1]. In osteoporosis (OP), the progressive reduction in bone mineral density and architectural deterioration leads to an increased risk of fragility fractures. The majority of hip fractures (up to 95%) happen as consequence of a fall [2] and are particularly severe, as they are associated with a 20% mortality rate within a year and high management costs. Therefore, preventing hip fractures and developing effective osteoporosis treatments is of paramount importance [1, 3–5].

Existing interventions to prevent hip fractures primarily include lifestyle interventions and pharmacological treatments. Lifestyle changes such as weight-bearing exercise are recommended to strengthen bones and muscles [6], while fall prevention strategies such as balance training and home safety assessments may reduce the risk of falls [7]. Calcium and vitamin D supplements are commonly prescribed to support bone health [8]. Pharmacological treatments include bisphosphonates, such as alendronate and risedronate [9–11], parathyroid hormone analogues, such as teriparatide [12], RANKL inhibitor (denosumab) [13], sclerostin inhibitors such as romosozumab [14], calcitonin analogues [15], selective oestrogen receptor modulators (SERMs) such as raloxifene [16, 17].

Currently, randomised clinical trials (RCTs) are the gold standard for evaluating the efficacy and safety of new treatments [18–20]. Placebo-controlled clinical trials are methodologically preferable, due to the presence of an internal control and the relatively smaller sample size required to demonstrate treatment effectiveness [21]. On the other hand, placebo-controlled clinical trials present ethical concerns, when effective treatments are available, especially in vulnerable elderly individuals at a high risk of fracture, who are deprived of potentially beneficial interventions [21]. Possible alternatives include the recruitment of individuals at low risk or the use of active control in place of the placebo group. For both cases, it has been reported that the required sample sizes would be prohibitive, due to the small difference in fracture incidence between the trial arms [21]. Therefore, there is a pressing need to develop alternative approaches that can improve drug development by complementing and/or replacing traditional clinical trials. As an example, among these the SABRE (Study to Advance BMD as a Regulatory Endpoint) project aims to reduce the length and cost of clinical trials by qualifying changes in bone mineral density (BMD) as a surrogate non-invasive endpoint for fractures in osteoporosis drug trials [22].

Computational tools for the design and assessment of biomedical products, i.e. *In Silico* Clinical Trials (ISCTs), are also emerging as promising alternatives [23]. ISCTs leverage computational models to simulate an intervention of interest in a virtual cohort, representative of the population of interest, enabling the testing of various interventions and hypotheses. Potential advantages associated with the use of ISCTs include the opportunity to test multiple interventions in the same cohort, to improve clinical trial design, to test treatments without subjecting patients to potential risks associated with clinical trials, to increase sample size (*In Silico*-augmented clinical trials), and to reduce the required time and costs [23–25].

Subject-specific Finite Element (FE) models of the human femur based on Computed Tomography (CT) scans have been extensively validated [26–28], and excellent accuracy in predicting failure load in a wide range of loading conditions has been reported (Standard Error of Estimate, SEE = 15% for failure load, SEE = 7% for strains, R^2^ = 0.89 between FE predictions and experimental measurements) [29–31]. Additionally, in a recent study [32] a multiscale model of side fall has been developed and applied to predict subject-specific current absolute risk of fracture (ARF0). Various stochastic parameters are used to simulate a wide range of falling scenarios, and fracture probability is obtained by comparing the impact force associated with multiple fall events with an FE-based failure load map. This probability, combined with the fall rate, is used to calculate ARF0. In a retrospective cohort, ARF0 had superior stratification accuracy compared to areal bone mineral density (aBMD) to stratify fractured vs. non-fractured patients [32]. ARF0 could potentially be used as a surrogate endpoint for osteoporosis phase I or phase II clinical trials in place of aBMD, after the required prospective clinical validation and qualification [33].

Currently, in phase III clinical trials regulatory bodies require fracture incidence as primary endpoint to evaluate and demonstrate the efficacy of OP treatments, as a more clinically relevant outcome [34, 35]. In our group, we are developing an ISCT technology (*BoneStrength*), based on a Markov Chain algorithm, to simulate the equivalent of a phase III clinical trial *In Silico,* able to predict fracture incidence as the final endpoint. La Mattina et al., (2023) developed a method to generate a virtual cohort of proximal femurs from a statistical atlas and demonstrated that the virtual cohort was representative of the physical cohort used to build the atlas, in terms of baseline aBMD and fracture risk (ARF0) [36]. In this study, the method was used to generate two virtual cohorts and replicate the placebo arms of two concluded clinical trials using *BoneStrength*. Fall events were stochastically sampled from a Poisson distribution, the multiscale fall model was used to estimate the impact load, and FE models to assess femur strength. Predicted fracture incidence was compared with the clinical data. This retrospective validation represents a mandatory step in the development of an ISCT technology to assess the efficacy of OP treatments, using fracture incidence as the predicted endpoint.

The aim of this study was to evaluate the accuracy of *BoneStrength* in predicting fracture incidence in two virtual populations of postmenopausal women, whose baseline characteristics replicated those reported for the placebo arms of two concluded clinical trials, by comparing the model predictions against the clinical outcome.

## Materials and Methods

In this study, we replicated the placebo arms of two clinical trials reported in the literature using the *In Silico* trial methodology developed in our group (*BoneStrength*). We generated virtual cohorts of Finite Element models of proximal femurs, by matching the baseline characteristics of the populations recruited in the studies. Subsequently, fracture incidence was predicted for both cohorts using a Markov Chain process. A Poisson distribution was used to model the probability of fall events, while a multiscale model was applied to simulate side falls and predict impact load. Fractures were predicted by comparing impact load with failure load associated with each fall event, obtained with subject-specific FE models. Three years of follow-up were simulated for both cohorts to estimate hip fracture incidence and compare the results with the clinical data.

### Clinical Data for Retrospective Validation

Two large concluded clinical trials from the literature were selected: the Long-term Intervention on Fractures with Tibolone (LIFT) and Fracture Reduction Evaluation of Denosumab in Osteoporosis Every 6 Months (FREEDOM) [37, 38]. Selection criteria were based on the population of interest (postmenopausal women, T-score < − 1, average age > 55 years), the presence of a placebo arm, and reported hip fracture incidence. Details of the baseline characteristics of the cohorts recruited in the selected trials and fracture incidence are reported in Table 1.

**Table 1:** Baseline characteristics of the populations recruited in the reference studies (placebo groups)

	Cummings et al., 2008 (LIFT)	Cummings et al., 2009 (FREEDOM)
Total Hip aBMD [g/cm^2^]	0.722 ± 0.096	0.709 ± 0.099
Age [yrs]	68.2 ± 5.2	72.3 ± 5.2
BMI [kg/m^2^]	25.7 ± 3.4	26.0 ± 4.2
Number of participants	2257	3906
Follow-up duration [yrs]	3	3
Number of hip fractures	14	43

We generated two virtual cohorts of proximal femurs to match the baseline characteristics of the placebo arms in terms of Total Hip aBMD.

### Virtual Cohort

The procedure applied to generate the virtual cohorts has been described in detail in La Mattina et al., 2023 [36] and is shortly summarised here. Briefly, a dataset of 94 CT-based FE-models of human femurs with high quality 10-node tetrahedral meshes (ANSYS ICEM CFD 2019R2, ANSYS Inc., USA) was used to generate a statistical anatomy atlas through principal component analysis (PCA). The original cohort was recruited in the context of a retrospective study, described in detail in Yang et al., 2014 [39]. The statistical atlas included both geometry (coordinates of nodes and reference system points) and density (density-based element-specific Young’s moduli) features. By sampling the inverse cumulative functions associated with each principal component, 93 stochastic variables following the same distributions were obtained, which were combined to generate both geometry and material property distribution for virtual femurs. To estimate the knee rotation centre position, proximal femur models were fitted with an anatomy atlas [40]. The procedure was implemented in MATLAB (MATLAB 2022a, MathWorks Inc., USA).

We generated 1270 and 1249 synthetic femurs to match the cohorts recruited in the LIFT and the FREEDOM trials, respectively. Virtual patients were progressively sampled from the distributions of principal components and total hip aBMD was calculated for each. Subsequently, patients were included in the virtual cohort or discarded until the population with the target numerosity and statistical distribution of total hip aBMD was obtained. From the two virtual cohorts, 32 and 24 models were respectively excluded due to convergence issues or excessive element distortion. Subject-specific height and weight were determined for each virtual patient based on their correlation with DXA data obtained using a linear regression [36], with a Pearson’s correlation coefficient of 0.4 was between height, weight, and femoral neck aBMD. The number of virtual patients was chosen to reproduce the typical sample size of phase III clinical trials, in the order of more than 1000 patients [18, 21, 41] while reducing the computational resources required for simulation.

### Fall Model

The impact force associated with each fall was determined with a multiscale model, as described in Bhattacharya et al. 2019 [32]. Briefly, the model estimates the force transmitted to the femur during a side fall by considering both subject-specific and stochastic parameters, which are sampled from probability distributions appropriately defined for the population of interest. Three models are included: a mechanistic body-floor impact model to compute peak impact force; a ground-skeleton force transfer model; a subject-specific FE model. The ground-skeleton force transfer model is applied to attenuate the impact force, to account for the attenuation provided by the presence of soft tissues around the hip (subject-specific parameter), different flooring materials (e.g. carpets), and wearable devices (such as hip protectors) [32]. Subject-specific soft tissue thickness was derived from individual BMI using a linear relationship tailored to the reference population [42]. The first two models were implemented using standard Python libraries NumPy and SciPy. Lastly, the FE model (paragraph 2.4) is used to calculate the femur strength in each loading condition.

In this work, we have assumed that muscle contraction and subsequent stiffening of the dynamic system had a negligible effect on the impact force transferred from the ground to the bone, as it is very unlikely that elderly individuals would be able to significantly contract their muscles before the impact. Falls, which occur unexpectedly and unintentionally during daily activities, result in impacts within a time frame of 300–750 milliseconds [43–46]. Even though subjects can intervene in the dynamics of falls, it has been estimated that the initial 300-450 milliseconds are dedicated to recognizing and attempting to prevent the fall [43, 44, 46]. Additionally, in elderly individuals, there is a general increase in reaction time, especially in choice reaction tasks that involve selecting an appropriate response strategy [47–53]. Comparative studies have estimated that this difference is in the order of hundreds of milliseconds, particularly in experimental setups that involve secondary mental tasks and considering the delay in EMG activation of the lower limbs [52, 54, 55]. Therefore, in this study it was assumed that no or negligible muscle contraction could occur in the time frame of the fall event.

The occurrence of falls in the community-dwelling elderly population was modelled as the occurrence of discrete events following a Poisson distribution, which assumes:

- Equiprobability of the occurrence of an event for all sub-intervals.
- Possibility for an event to occur multiple times within the same sub-interval.
- Independence of events occurring in different sub-intervals.

Under these assumptions, the probability that an event with an average probability of occurrence λ will occur *n* times is expressed by the equation:

$${P}_{\lambda }\left(\text{n}\right)= \frac{{\lambda }^{n}}{n!}{e}^{-\lambda }$$

We determined the number of falls per year for each patient by sampling the distribution (MATLAB 2022a, MathWorks Inc., USA). The value of λ = 0.65 falls/year was based on data from the literature for the population of interest, in terms of falls per person per year [56], obtained from concluded clinical trials where fall-assessment techniques of proven accuracy were applied [57–59]. Implicitly, it was assumed the strong hypothesis of non-variability of fall risk within the population, among different time intervals, and within the intervals themselves.

### FE Models

Subject-specific FE models were used to predict the biomechanical failure load associated with each fall event. Each FE model had a quadratic tetrahedral mesh with maximum element size of 3 mm and heterogeneous isotropic linear elastic material properties [60]. The impact force was applied at the centre of the femoral head. For each fall, the impact direction between the greater trochanter and the ground was stochastic and defined by two angles, namely the intra-extra rotation (α) and abduction-adduction (β) angles of the femur. The intervals α = [−30°, 30°] and β = [0°, 30°] were discretized with a step of 1°, resulting in 1891 possible force directions. It was assumed:

- Equiprobability among the possible values of α and β.
- Reciprocal independence of α and β values.
- Independence of α and β from time, subject-specific factors, and the number of falls.

For each fall, a FE simulation was run by applying a 1000 N load in the centre of the femoral head with the associated orientation (ANSYS Mechanical APDL 2022a, ANSYS Inc., USA) [31]. This approach was used to predict direction-specific failure load, as in previous studies it has been reported that femur strength is strongly dependent on the impact angle [31]. Simulating multiple side fall directions also had a significantly better stratification accuracy compared with single-load strength in a retrospective cohort [31]. The distal diaphysis was coupled with a node in the centre of the knee joint with multi-point constraints, therefore the femur was allowed to rotate around an axis perpendicular to load direction through the knee centre. A rigid plane was positioned at the furthest point of the greater trochanter along the load direction and a non-linear frictionless contact between the femur surface and the rigid plane was applied [31].

Failure load was determined by calculating the load intensity required to generate a principal tensile strain of 0.73% or a principal compressive strain of 1.04% [61] (whichever occurred first) on the surface of the femur. Nodal principal strains were averaged over a sphere with a radius of 3 mm (NumPy and SciPy libraries, Python) [62]. This fragile fracture criterion has been previously validated, showing excellent prediction accuracy compared to experimental results obtained with cadaveric bones [61].

### Fracture Incidence

**Fig. 1:**
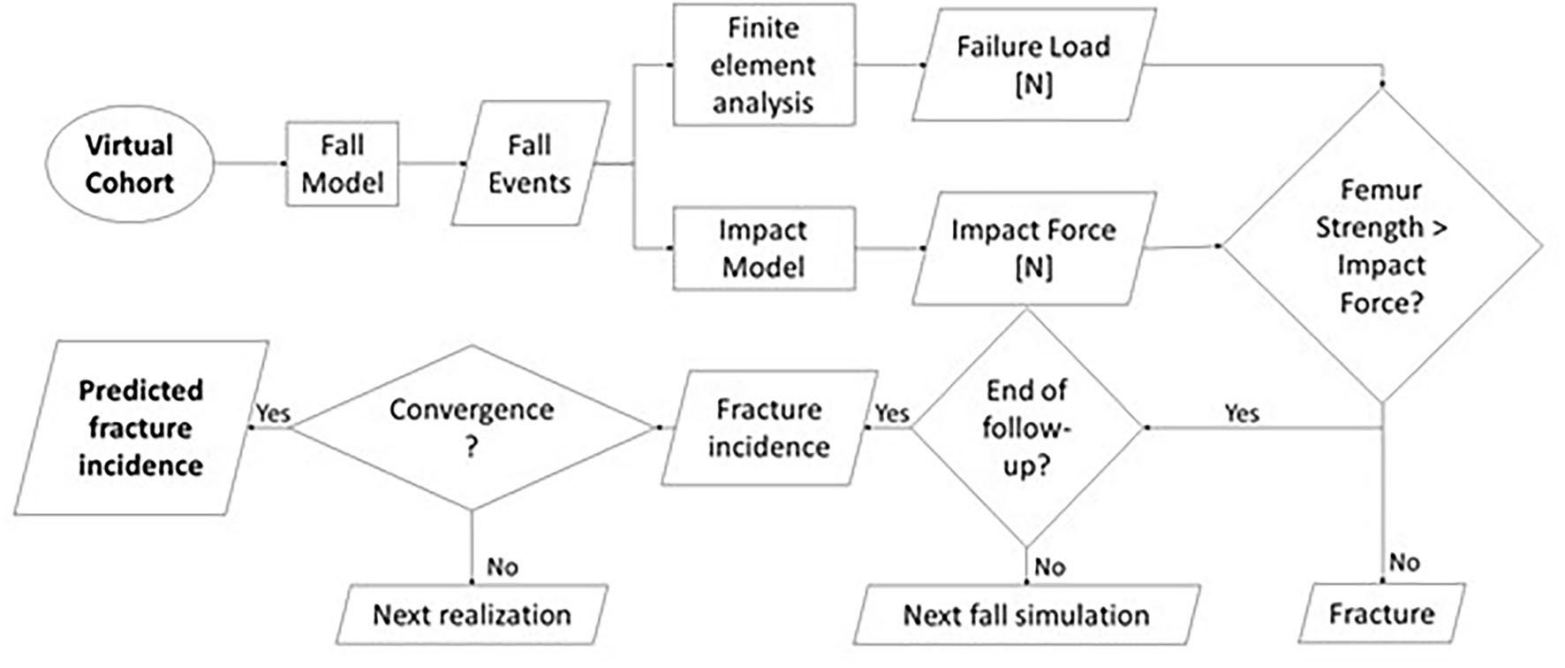
Markov-Chain algorithm architecture

The Markov Chain algorithm used to predict fracture incidence in the virtual cohort is reported in Figure 1. For each patient, fall events were sampled from the Poisson distribution, as described in paragraph 2.3. In the event of a fall, direction and impact load were stochastically sampled from the corresponding distributions, and the FE simulation was run to obtain failure load. Virtual patients were classified as fractured when the impact force exceeded the failure load in the impact direction and excluded from subsequent simulations. This, besides saving computational time, simulates the role of hip fracture as a clinical endpoint. Three years of follow-up were simulated with a time step of 1 year by iteratively sampling fall events, comparing the impact force with the corresponding failure load, and excluding fractured patients. The computed incidence of hip fractures over three years was compared with the clinical data.

For each virtual cohort, 10 realizations of the Markov Chain process were run, to reach convergence of the estimated fracture incidence. Each realization required approximately 2500 FE simulations (4 cores and 28 GB of RAM per simulation).

This pipeline is currently running on High-Performance Computing Infrastructure Ares (Cyfronet, Poland) through the Model Execution Environment (MEE, https://mee.cyfronet.pl/).

### Statistical Analysis

A two-sample t-test (ttest2 function, MATLAB 2022a, MathWorks Inc., USA) was used to verify that the distributions of Total Hip aBMD and BMI (which are strongly correlated with hip fracture risk [63]) in the virtual and reference cohorts were not significantly different.

Convergence of the predicted fracture incidence obtained with the Markov Chain algorithm was assessed by evaluating the mean number of fractures as a function of the number of realizations. Bootstrapping was employed to determine the relative difference in the mean obtained by including an additional realization. A relative difference of 1% was used as threshold to define convergence (NumPy and SciPy libraries, Python).

## Results

**Fig. 2:**
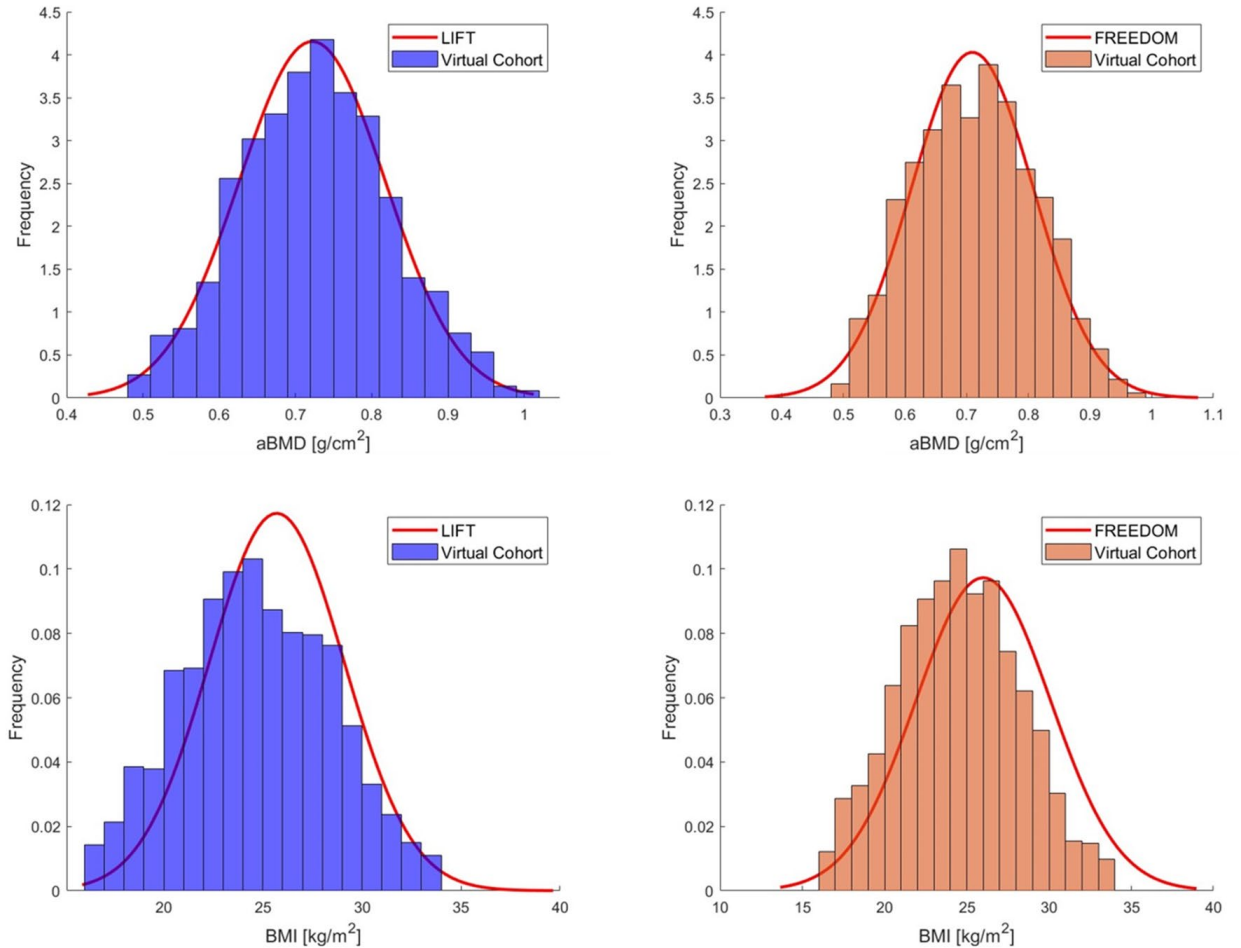
Distributions of Total Hip aBMD and BMI in the virtual cohorts compared with clinical data reported in the reference studies

The distributions of Total Hip aBMD in the two virtual cohorts were not significantly different compared to the cohorts recruited in the LIFT study (t-test, p = 0.31) and the FREEDOM study (p = 0.38). BMI distributions were significantly different between virtual cohorts and clinical data for both studies (p < 0.001). A comparison between the distributions of Total Hip aBMD and BMI in the two virtual cohorts and the reference populations is reported in Figure 2.

Fracture incidence predicted by the model converged after 10 realizations (relative difference of cumulative mean = 0.36% for LIFT, and 0.32% for FREEDOM).

For the virtual cohort based on the LIFT study (N = 1238 virtual patients), 12 ± 2 fractures in three years were predicted. For the virtual cohort based on the FREEDOM study (N = 1225) 18 ± 4 hip fractures were obtained over a simulated follow-up of three years. Minimum and maximum number of fractures predicted across all realisations are reported in Table 2.

**Table 2:** Number of fractures predicted for the two virtual cohorts in three years follow up: average and SD, minimum and maximum across all realisations

	Predicted fracture incidence (Mean ± SD)	Min predicted fracture incidence	Max predicted fracture incidence	Reported fracture incidence [37, 38]
LIFT (N = 1238)	12 ± 2	8	15	7.7 (14 in 2257 patients)
FREEDOM (N = 1225)	18 ± 4	13	25	13.5 (43 in 3906 patients)

## Discussion

In this study, the *In Silico* trial methodology *BoneStrength* was applied to predict the hip fractures in two simulated cohorts of postmenopausal women, designed to replicate the baseline characteristics of participants recruited in the placebo arms of two concluded clinical trials. Predicted fracture incidence was compared to the clinical data reported in the studies to assess the model predictive ability.

The range of hip fracture incidence predicted by the model was consistent with the clinical data reported in both studies, highlighting the approach was able to adequately model the phenomenon of interest; nevertheless, on average the number of fractures was consistently overestimated compared with the clinical data. This could be linked to the simplified approach applied to model the frequency and dynamics of falls, as discussed in the following paragraphs. The occurrence of hip fractures in a given cohort depends on several factors such as the frequency of fall events, which may be influenced by external factors (e.g. presence of fall hazards), the ability of the patient to prevent or attenuate the fall, the characteristics of the impact surface, and more. In our model these are represented with a probability distribution associated with each phenomenon; in this perspective, a clinical trial can be considered as one observation (or realisation) of the process outcome, while more than one trial would be needed to collect data about its variability.

The envisioned application for this *In Silico* trial technology is to support the assessment of treatment efficacy. In this context, a key strength is the possibility to predict fracture incidence, which is the clinical endpoint required in phase III by regulatory authorities, in a representative population of interest. The stochastic approach also allows to model the complexity and variability of the fall and fracture phenomena. Nevertheless, limitations linked to the generation of virtual cohorts and to the fall model are discussed in the following paragraphs.

In this study, the approach used to model propensity to fall in the population was simplified; it was assumed that the underlying probability mass function associated with *n fall events* was equal for all virtual patients. Nevertheless, propensity to fall is not homogeneous among subjects, with approximately 30% of subjects experiencing one or more falls per year and 70% of non-fallers [37]. Additionally, fall rate is strongly correlated with older age, general frailty (e.g. dynapenia, presence of comorbidities, equilibrium and postural impairment) and lower education level [64]. Similarly, a previous fall is a significant risk factor for further falls and is associated with fear of falling in the elderly [65]; therefore, assuming the independence of subsequent events also represents a limitation of the current approach. Future developments will be focused on including inter-subject variability in fall rate, e.g. by integrating rule-based predictors based on subject-specific risk factors [66]. Lastly, it is important to highlight that the value of λ (average fall rate) used in this study was based on data from a systematic review reported for the population of interest, in terms of falls per person per year [56]. In this systematic review, concluded clinical trials that performed fall assessment using techniques of proven efficacy were selected [57–59]. The median fall rate was 0.65 falls/year, which differs deeply from the fall rate reported in the clinical trials replicated in this study (0.03–0.05 falls per person per year) [37, 38]. This difference may be due to the fact that falls were recorded as adverse events by clinicians; it has been shown that this approach may lead to an underestimation of recorded falls, given the tendency to record injury-related events, while potentially overlooking non-injurious falls [57, 58]. In this study, fall rate reported in Gillespie et al., (2012) [56] was considered more reliable and applied to model fall incidence.

Likely, a correlation also exists between fall frequency and ability to attenuate a fall, as ageing and general frailty may affect not only the frequency but also the dynamics of falls, although their dependence is not trivial given the lack of a clear relationship [32, 65, 67–69]. For example, incorrect weight shifting has been suggested as the main cause for falling in the elderly [45, 70], which likely produces a significant population-specific difference in fall dynamics, and therefore in impact velocity and force, correlated with age and frailty. In this study, clinical trials cohorts were replicated by matching the baseline aBMD distributions. Implicitly, we assumed that age does not affect postural parameters, although this is a simplification. In fact, predicted fracture incidence was less overestimated for the virtual cohort replicating the FREEDOM cohort, which was on average older compared to the one enrolled in the LIFT study; 73.6% of subjects were older than 70 years of age and 31.6% older than 75; on the other hand, in the LIFT study only 40% of subjects were older than 70 [37, 38]. This discrepancy suggests that the model could be improved by including the effect of frailty, for example on postural reflex, considering that in older cohorts a cognitive delay would result in lower attenuation of the impact force upon falling.

The anatomy atlas used to generate virtual cohorts was based on data from a retrospective study [39] to obtain geometry and BMD distribution. Other subject-specific parameters (height, weight, soft tissue thickness) were derived from either internal correlations with other parameters or from literature data reported for similar populations. While this approach allows to introduce the population variability in the model to some extent, it is dependent on the amount and quality of data available; additionally, depending on the application, it might be necessary to adapt some of the parameter distributions to model different populations (e.g. ethnicity).

As reported previously, the Body Mass Index (BMI) distributions associated with the virtual cohorts were significantly different from those reported in the reference studies. Our model generates height and weight values for each virtual model based on the relative correlations with BMD obtained from the physical cohort [36]. In both virtual cohorts, the individual correlations between bone mineral density and BMI align with existing literature [45, 71]. More importantly, no significant variation in hip fracture risk has been reported for the corresponding range of BMI [63]. Therefore, this difference is not expected to have an impact on predicted fracture incidence and was considered acceptable.

Another limitation of the study is that no osteoporosis progression was modelled, by assuming that FE material properties were constant over the simulated follow-up years. While bone loss may increase fracture incidence due to structural weakening, nevertheless our preliminary simulations indicate that, in the relatively short follow-up period of the reference studies (three years) [37, 38], a decrease in BMD had limited effect on predicted fracture incidence. Therefore, in first approximation this parameter was kept constant and will be included in the model in future developments, while this work was focused on the algorithm to predict hip fractures in the population of interest and replication of concluded clinical trials to assess its accuracy.

Lastly, the aim of this model was the prediction of hip fractures only, while fractures at other anatomical sites (e.g. pelvic) may occur. Despite this limitation, hip fractures are the second most common clinical endpoint for evaluating the efficacy of osteoporosis treatments, following vertebral fractures [21].

A future development we are exploring is the possibility to use a machine learning-based surrogate model for predicting failure load in multiple fall directions, which could potentially replace the FE simulations. If appropriately validated, this model would allow to significantly reduce the computational cost of the *In Silico* trial simulation.

*BoneStrength* represents the first *In Silico* Clinical Trial methodology for the development and assessment of interventions to reduce hip fractures. This technology can potentially be used in the future to improve the time- and cost-effectiveness of drug development in osteoporosis and to produce evidence on treatments’ efficacy for preventing hip fractures in postmenopausal women. Potential applications include the improvement of clinical trial design and de-risking, e.g. by reducing the required sample size and/or partially replacing human experimentation, which could also potentially overcome the ethical concerns around the inclusion of high-risk patients in placebo-controlled trials [19, 21].

In conclusion, the *In Silico* trial technology *BoneStrength* was applied for the replication of the placebo arms of two clinical trials on OP treatments, showing its potential for predicting hip fracture incidence. The model refinement and validation will be carried out for application in the drug development process and assessment.

## Data Availability

Model data are available in the University of Bologna institutional repository under terms of CC BY 4.0 International license at the link:10.6092/unibo/amsacta/7934.
